# Selective HDAC Inhibition for the Disruption of Latent HIV-1 Infection

**DOI:** 10.1371/journal.pone.0102684

**Published:** 2014-08-19

**Authors:** Kirston M. Barton, Nancie M. Archin, Kara S. Keedy, Amy S. Espeseth, Yan-ling Zhang, Jennifer Gale, Florence F. Wagner, Edward B. Holson, David M. Margolis

**Affiliations:** 1 Department of Microbiology and Immunology, University of North Carolina at Chapel Hill, Chapel Hill, North Carolina, United States of America; 2 Department of Medicine, University of North Carolina at Chapel Hill, Chapel Hill, North Carolina, United States of America; 3 Department of Epidemiology, University of North Carolina at Chapel Hill, Chapel Hill, North Carolina, United States of America; 4 Department of Antiviral Research, Merck Research Laboratories, West Point, Pennsylvania, United States of America; 5 Stanley Center for Psychiatric Research, Broad Institute of Massachusetts Institute of Technology and Harvard University, Cambridge, Massachusetts, United States of America; Vita-Salute San Raffaele University School of Medicine, Italy

## Abstract

Selective histone deacetylase (HDAC) inhibitors have emerged as a potential anti-latency therapy for persistent human immunodeficiency virus type 1 (HIV-1) infection. We utilized a combination of small molecule inhibitors and short hairpin (sh)RNA-mediated gene knockdown strategies to delineate the key HDAC(s) to be targeted for selective induction of latent HIV-1 expression. Individual depletion of HDAC3 significantly induced expression from the HIV-1 promoter in the 2D10 latency cell line model. However, depletion of HDAC1 or −2 alone or in combination did not significantly induce HIV-1 expression. Co-depletion of HDAC2 and −3 resulted in a significant increase in expression from the HIV-1 promoter. Furthermore, concurrent knockdown of HDAC1, −2, and −3 resulted in a significant increase in expression from the HIV-1 promoter. Using small molecule HDAC inhibitors of differing selectivity to ablate the residual HDAC activity that remained after (sh)RNA depletion, the effect of depletion of HDAC3 was further enhanced. Enzymatic inhibition of HDAC3 with the selective small-molecule inhibitor BRD3308 activated HIV-1 transcription in the 2D10 cell line. Furthermore, *ex vivo* exposure to BRD3308 induced outgrowth of HIV-1 from resting CD4+ T cells isolated from antiretroviral-treated, aviremic HIV+ patients. Taken together these findings suggest that HDAC3 is an essential target to disrupt HIV-1 latency, and inhibition of HDAC2 may also contribute to the effort to purge and eradicate latent HIV-1 infection.

## Introduction

The persistence of latent human immunodeficiency virus type 1 (HIV-1) infection, despite highly effective antiretroviral therapy (ART), poses a formidable obstacle to eradication of HIV-1. This reservoir of quiescent HIV-1 proviruses is established early during acute infection and persists in long-lived resting CD4+ T cells throughout the life of an infected individual [1]–[3]. Millions of people are newly infected with HIV-1 each year, and the health and economic costs of life-long antiretroviral regimens are a heavy burden. Therefore, approaches to eradicate HIV-1 are needed [4]. A better understanding of the factors that establish and maintain HIV-1 latency will allow the design and testing of specific, selective therapeutic eradication strategies.

Resting CD4+ T cells are resistant to productive HIV-1 infection due to the quiescent phenotype of these cells, which is characterized by low nuclear levels of the cellular transcription factors that are required for viral expression [5]–[8]. Although evidence exists that HIV-1 occasionally overcomes these barriers and directly infects resting CD4+ T cells, the latent resting cell reservoir is primarily thought to be generated when an activated CD4+ T cell is infected by HIV-1 as it transitions to the long-lived, resting memory CD4+ T cell state [9], [10]. Once an HIV-1 provirus has integrated into the host's genome, the virus can enter a quiescent state that is able to persist in the presence of ART. Furthermore, replication-competent virus can be recovered from latently infected CD4+ T cells following mitogen stimulation or exposure to agents such as HDAC inhibitors or protein kinase agonists [11], [12].

During latency, multiple restrictive factors are associated with the HIV-1 long terminal repeat (LTR) promoter, blocking efficient transcriptional initiation and mRNA elongation. Among these factors are HDACs, which are a family of enzymes that regulate transcription of numerous cellular and viral genes by removing acetyl groups from the lysine residues on both histones and non-histone proteins [13], [14]. Deacetylation of histone tails results in removal of important docking signals that are required for binding of activating transcription factors. The result is an overall repressive transcriptional environment. HDACs are divided into four classes based upon their amino acid sequence, domain organization, and catalytic dependence on zinc (Class I, II, and IV) or nicotinamide adenine dinucleotide (NAD+) (Class III) [15]. The class I HDACs include HDAC1, −2, −3, and −8, while HDAC4, −5, −6, −7, −9, and −10 make up the class II HDACs, and HDAC11 is the sole member of class IV. Class III HDACs include sirtuins 1–7, which are NAD+-dependent deacetylases that are structurally unrelated to the other HDACs. Class III HDACs have not been associated with maintenance of HIV-1 latency and are not sensitive to the type of HDAC inhibitors that induce HIV-1 expression. Therefore, this study primarily focused on the role that the Class I HDACs play in HIV-1 expression.

Non-selective and class I-selective HDAC inhibitors are potent inducers of HIV-1 expression in both cell line models of HIV-1 latency and in *ex vivo* outgrowth assays using resting CD4+ T cells from HIV-1-infected individuals [11], [16]–[19]. Furthermore, the HDACi SAHA upregulates expression of cell-associated HIV-1 RNA in the resting CD4+ T cells of ART-treated, aviremic patients *in vivo* [20]. The class I HDACs, HDAC1, −2, and −3, are recruited to the HIV-1 LTR in cell line models of HIV-1 latency [14], [21]–[24]. These class I HDACs are highly expressed in the nuclei of resting CD4+ T cells, and sub-class I selective inhibitors for HDAC1, −2, and −3 are strong inducers of latent HIV-1 expression in resting CD4+ T cells [21]. However, inhibitors selective for the class II HDACs do not induce expression of HIV-1 [21]. HDAC4, −6, or −7 have not been demonstrated to directly bind to the HIV-1 LTR [21]. Inhibitors that act only on HDAC1 and −2 but do not inhibit HDAC3 do not activate latent HIV-1 [21]. This data suggests that HDAC3 enzymatic inhibition is crucial for induction of expression from quiescent HIV-1 proviruses, but the contributions of HDAC1 and −2 are unclear. Determining the minimal set of HDACs that must be inhibited to induce latent HIV-1 expression may focus efforts to identify and develop isoform selective HDAC inhibitors; such inhibitors might have fewer effects on other cellular genes and less clinical toxicity.

In an effort to better understand the role of individual class I HDACs in regulation of HIV-1 transcription, we chose to explore the impact of single or combination shRNA-mediated depletion of HDAC1, −2, and −3 on HIV-1 expression in a T cell line model of latency.

## Materials and Methods

Study participants provided written informed consent under a protocol that was approved by the UNC Biomedical Institutional Review Board.

### Cell lines

2D10 cells were provided by the lab of Jonathan Karn [27]. The 2D10 cells contain a HIV-1 genome with GFP inserted in the place of nef. J89 and THP89 cells were kindly provided by Dr. David Levy and contain a full length HIV-1 genome with GFP inserted between env and nef [25]. J-Lat 6.3 cells were acquired from the NIH AIDS Reagent Program, Division of AIDS, NIAID, NIH, from Dr. Eric Verdin and contain a full length HIV-1 genome with a frameshift in env that restricts the insert from producing env or nef [26]. All cell lines were cultured in RPMI 1640 with 10% FBS, 100 U/ml penicillin (Invitrogen), and 100 µg/ml streptomycin (Invitrogen) [27]. All experiments were performed using cells that had been passaged fewer than ten times. Cell cultures were maintained at 37°C under 5% CO_2_.

### Flow cytometry analysis

First, the cells were washed once in PBS and then fixed in PBS containing 3.2% paraformaldehyde. Flow cytometry was performed using an Attune flow cytometer (Applied Biosystems, Carlsbad, CA). Analysis of GFP expression was performed using FlowJo software (Tree Star in.; Ashland, OR), and the statistical analysis was performed using Graphpad Prism software (La Jolla, CA). The results are shown as the mean of at least three independent experiments, and the error bars indicate the standard error of the mean (SEM).

### Transduction of shRNAs

Three million 2D10 cells were aliquoted into fresh media 24 h prior to transduction. Then, lentiviruses containing shRNAs specific to the individual HDACs were added to the 2D10 cells, and 2 µg/ml puromycin was added to the cells 24 h after addition of lentiviruses to select for cells containing the shRNA expressing vectors. In experiments that required drugs to be added to the cells, the drugs were added at 72 h post-transduction. At 96 h post-transduction, cell samples were collected for flow cytometry and mRNA expression analysis. Independent transductions were performed for each experiment. The values shown are the mean of at least three independent experiments, and the error bars indicate the standard error of the mean.

### HDAC inhibitors

2D10 cells that had been depleted of HDAC1, −2, or −3 with shRNAs were incubated with the indicated HDAC inhibitors for 18 h. Merck 12 was used at a concentration of 20 µM, Merck 13 was used at a concentration of 200 nM, and SAHA was used at a concentration of 250 or 500 nM. Mrk12, Mrk13, and SAHA were generously provided by Merck Research laboratories (West Point, PA). TSA (T8552, Sigma, St Louis, MO) was used at a suboptimal concentration of 25 nM, which stimulates detectable GFP in only 5% of 2D10 cells whereas 150 nM stimulates detectable GFP in over 85% of 2D10 cells. Droxinostat (S1422, Selleckchem.com, Houston, TX) was used at a concentration of 2 µM. BRD3308 was synthesized at the Broad Institute at >95% purity [28].

2D10 cells were treated with BRD3308 and then washed and resuspended in fresh media. The cells were then kept in tissue culture for 24 hours and then collected, fixed, and analyzed using flow cytometry.

### Quantitative viral outgrowth assay (QVOA)

This assay of the frequency of replication-competent HIV-1 within ART-suppressed patient's resting CD4+ T cells has previously been described in detail [29]. Study participants provided written informed consent under a protocol that was approved by the Institutional Review Board. Briefly, four patients (Pt 1–4) donated peripheral blood mononuclear cells via leukopheresis, and resting CD4+ T cells were purified using negative selection. The cells were incubated with antiretroviral drugs to prevent spread of infection prior to limiting dilution co-culture. BRD3308 (15 uM) or SAHA (335 nM) was added to aliquots of resting CD4+ T cells which were then plated in limiting dilution replicates of 2.5 or 1 million, 0.5 million, and 0.1 million cells and incubated at 37°C under 5% CO_2_. Drugs were removed from the cultures after 24 hours. PBMCs from an uninfected donor were added to the cultures twice, and viral outgrowth was measured using p24 ELISA on the supernatant collected on day 15, and p24-positive cultures verified on day 19. The results are presented as the infectious units per million (IUPM) resting CD4+ T, calculated using a maximum likelihood method. For Patient 3, an IUPM value from the PHA condition was is not available; therefore, the IUPM for PHA shown represents the pooled data of three prior determinations from this patient.

### HDAC inhibition assay (microfluidic lab-on-a-chip assay)

All HDACs were purchased from BPS Bioscience. Compounds were tested in duplicate in a 12-point dose curve with 3-fold serial dilution starting from 33 µM. Purified HDACs were incubated with 2 µM carboxyfluorescein (FAM)-labeled acetylated peptide substrate and test compound for 60 min at room temperature, in HDAC assay buffer that contained 50 mM HEPES (pH 7.4), 100 mM KCl, 0.01% BSA, and 0.001% Tween-20. Reactions were terminated by the addition of the known pan HDAC inhibitor LBH-589 (panobinostat) at a final concentration of 1.5 µM. Substrate and product were separated electrophoretically, and the fluorescence intensity in the substrate and the product peaks were determined and analyzed by Labchip EZ Reader. The reactions were performed in duplicate for each sample. IC_50_ values were automatically fitted by Genedata Screener software using 4-parameter logistic dose response model [30].

### RNA extraction and quantitative RT-PCR

RNA was extracted from cells using a QIAgen RNeasy Mini Kit (Valencia, CA) following the manufacturer's protocol. DNA was removed from RNA extracts by DNAse digestion (Promega; Madison, WI), and cDNA was synthesized using the SuperScript III First-Strand Synthesis for RT-PCR kit from Invitrogen. Quantitative PCR was performed on cDNA with a Bio-Rad CFX96 or CFX384 using QuantiTect Multiplex PCR Mastermix (QIAgen) and the following primer pairs and 5′ FAM-labeled probes: HDAC1 5′ TGAGGACGAAGACGACCCT (forward), 5′ CTCACAGGCAATTCGTTTGTC (reverse), and 5′ CAAGCGCATCTCGATCTGCTCCTC (probe) [31]; HDAC2 5′ CTTTCCTGGCACAGGAGACTT (forward), 5′ CTCATTGGAAAATTGACAGCATAGT (reverse), and 5′ AGGGATATTGGTGCTGGAAAAGGCAA (probe); and HDAC3 5′ GGTGGTTATACTGTCCGAAATGTT (forward), 5′ GCTCCTCACTAATGGCCTCTTC (reverse), and 5′ AGCAGCGATGTCTCATATGTCCAGCA (probe). Expression of GFP mRNA from the HIV-1 promoter was measured using the primers 5′ GGAGCGCACCAT CTTCTTCA(forward) and 5′ AGGGTGTCGCCCTCGAA reverse along with the 5′ FAM labeled probe 5′ CTACAAGACCCGCGCCGAGGTG. A glyceraldehyde 3-phosphate dehydrogenase (GAPDH) primer pair and 5′ HEX-labeled probe were included with each reaction for normalization: 5′ GCACCACCAACTGCTTAGCACC (forward), 5′ TCTTCTGGGTGGCAGTGATG (reverse), and 5′ TCGTGGGAAGGACTCATGACCACAGTCC (probe) [32]. Relative mRNA expression was calculated using the 2^−ΔΔct^ method. The data shown is the mean of at least three independent experiments, and the error bars represent the standard error of the mean.

### Cell proliferation assays

Cellular proliferation and viability of the 2D10 cells were determined 96 hours post transduction using the CellTiter-blue cell viability assay (Promega; Madison, WI) according to the manufacture's instructions. The assay was read using a Spectramax M3 microplate reader (Molecular devices, Sunnyvale, CA) at fluorescence 560/590 nM. Viability was calculated as the percent of viability relative to the scrambled shRNA condition. At least three independent experiments were performed for each condition. The values shown in the graphs represent the mean ± the SEM.

### Statistical analysis

Graphpad Prism software was used to analyze the data. The Student's t test was used to compare the mean GFP protein or mRNA expression in 2D10 cells following transduction with a scrambled shRNA to cells transduced with HDAC1, −2, and −3 shRNAs. Furthermore, the Student's t test was used to compare the mean GFP expression following incubation with HDAC inhibitors after transduction from at least three independent experiments. A p-value of less than .05 was considered statistically significant.

## Results

### HDAC3 negatively regulates HIV-1 expression in 2D10 cells

To determine the individual contribution of each HDAC to the maintenance of HIV-1 latency, we first evaluated isozyme-specific HDAC knockdowns in the 2D10 cell line model of HIV-1 latency. 2D10 cells contain a single, transcriptionally silent HIV-1 genome that is integrated into the cellular DNA and contains GFP in place of nef [27]. The 2D10 cell line was clonally selected for a low level of basal HIV-1 expression, but it is inducible by exposure to appropriate stimuli, such as tumor necrosis factor alpha (TNF-α) or HDAC inhibitors [27]. The selection of an appropriate model system is critical, as although resting CD4+ T cells are inducible following exposure to HDAC inhibitors *in vitro* and *in vivo* [20], [29], model systems in cell lines [33] or even primary cell model systems [34] are not uniformly responsive to HDAC inhibitors.

**Figure 1:**
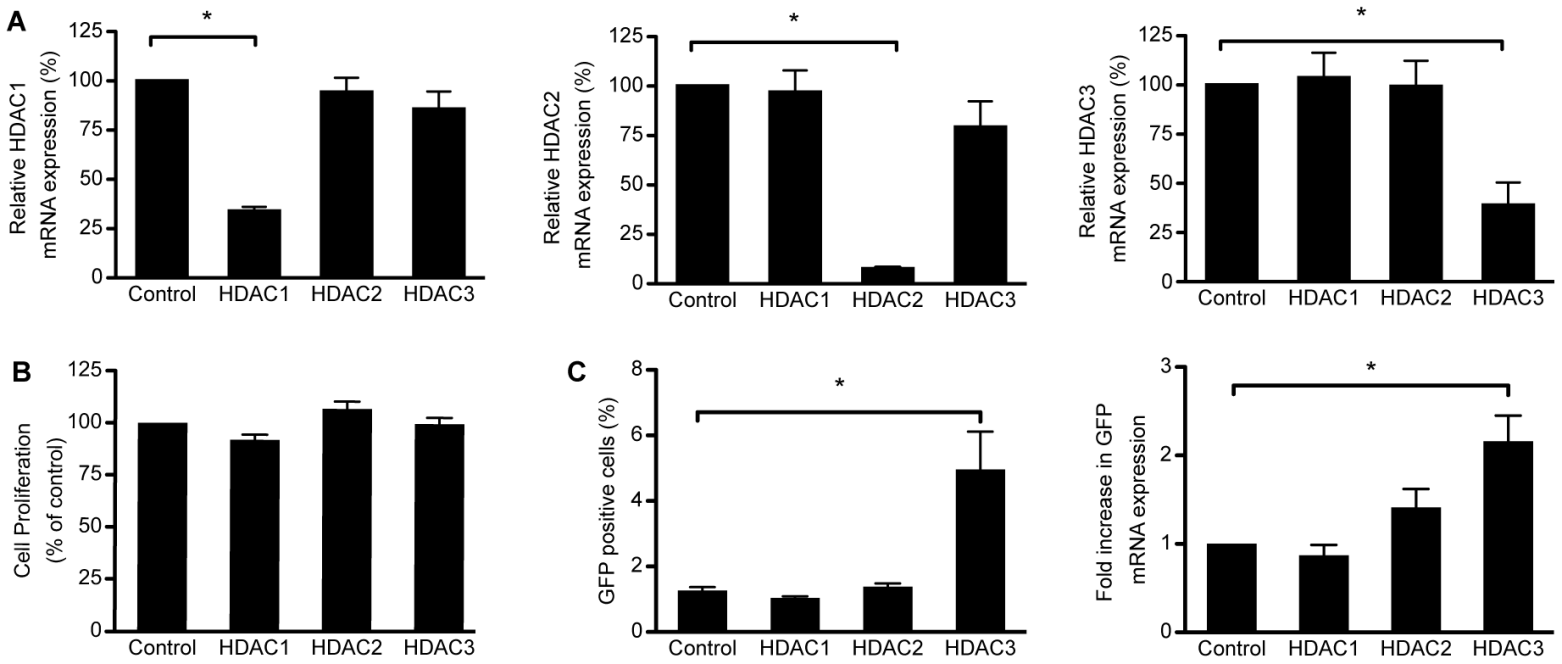
Depletion of HDAC3 significantly increases expression from the HIV-1 promoter. A. Expression of the indicated HDAC mRNAs following shRNA mediated depletion of HDAC1, −2, or −3 in 2D10 cells. Depletion of HDAC1, −2, or −3 was significant and specific for the targeted HDAC. B. Cell proliferation was not significantly affected following depletion of HDAC1, −2, or −3 in 2D10 cells. C. The percentage of 2D10 cells expressing GFP protein from the HIV-1 promoter following depletion significantly increased following depletion of HDAC3 in comparison to control cells transduced with the scrambled shRNA, but not following depletion of HDAC1 or −2. The same effect was seen when GFP mRNA expression was directly measured. Expression was not significantly altered from baseline following transduction with non-specific shRNA. (*p<0.05).

2D10 cells were transduced with a scrambled shRNA control sequence or with shRNAs targeting HDAC1, −2, or −3. The HDAC mRNA levels following knockdown with the shRNAs were compared to the expression levels from the scrambled shRNA control condition 96 h post-transduction. Following transduction, HDAC1, −2, and −3 mRNA levels were reduced by 70%, 92%, and 60%, respectively (Fig. 1A). The knockdowns were specific to the HDAC targeted (Fig. 1A). Furthermore, cellular viability was not affected by transduction with shRNAs targeting HDAC1, −2, or −3 when compared to cells that had been transduced with the scrambled shRNA control 96 h post-transduction (Fig. 1B).

Because transcription from the HIV-1 promoter is induced by HDAC inhibitors that target the class I HDACs 1, 2, and 3, we decided to determine if depletion of a single HDAC was sufficient to induce transcription from the HIV-1 LTR. Therefore, we assessed the impact of individual HDAC knockdown on HIV-1 expression in 2D10 cells by monitoring GFP protein and mRNA expression from the HIV-1 LTR for up to 96 hours after transduction. Depletion of HDAC3 in 2D10 cells led to a statistically significant increase in the percentage of cells expressing GFP protein and in mRNA expression from the HIV-1 promoter when compared to the scrambled shRNA control cells (Fig. 1C). However, individual HDAC1 or −2 knockdown did not induce a significant amount of HIV-1 LTR driven GFP protein or mRNA expression in 2D10 cells. A slight increase in GFP mRNA was observed following depletion of HDAC2 in the 2D10 cells. However, this change was not significant. The small change in mRNA expression that was observed in the cell population was not sufficient to effect a change in the percentage of cells positive for GFP protein expression indicating that the change may reflect a low level of expression throughout the population rather than a substantial increase in a subset of cells, which would have resulted in an increase in the number of GFP positive cells.

Using the same methodology as above, we also depleted the J89, J-Lat6.3, and THP89 cell line models of latent HIV of the individual HDACs and did not observe a significant increase in HIV expression from any of the conditions. This result may be due to variability between the different cell line models as observed in previous studies [34], [35]. This result further illustrates the importance of extending and validating findings in patient cells.

### Concurrent knockdown of HDAC1 or −2 with HDAC3 does not further increase expression from the HIV-1 promoter compared to depletion of HDAC3 alone

**Figure 2:**
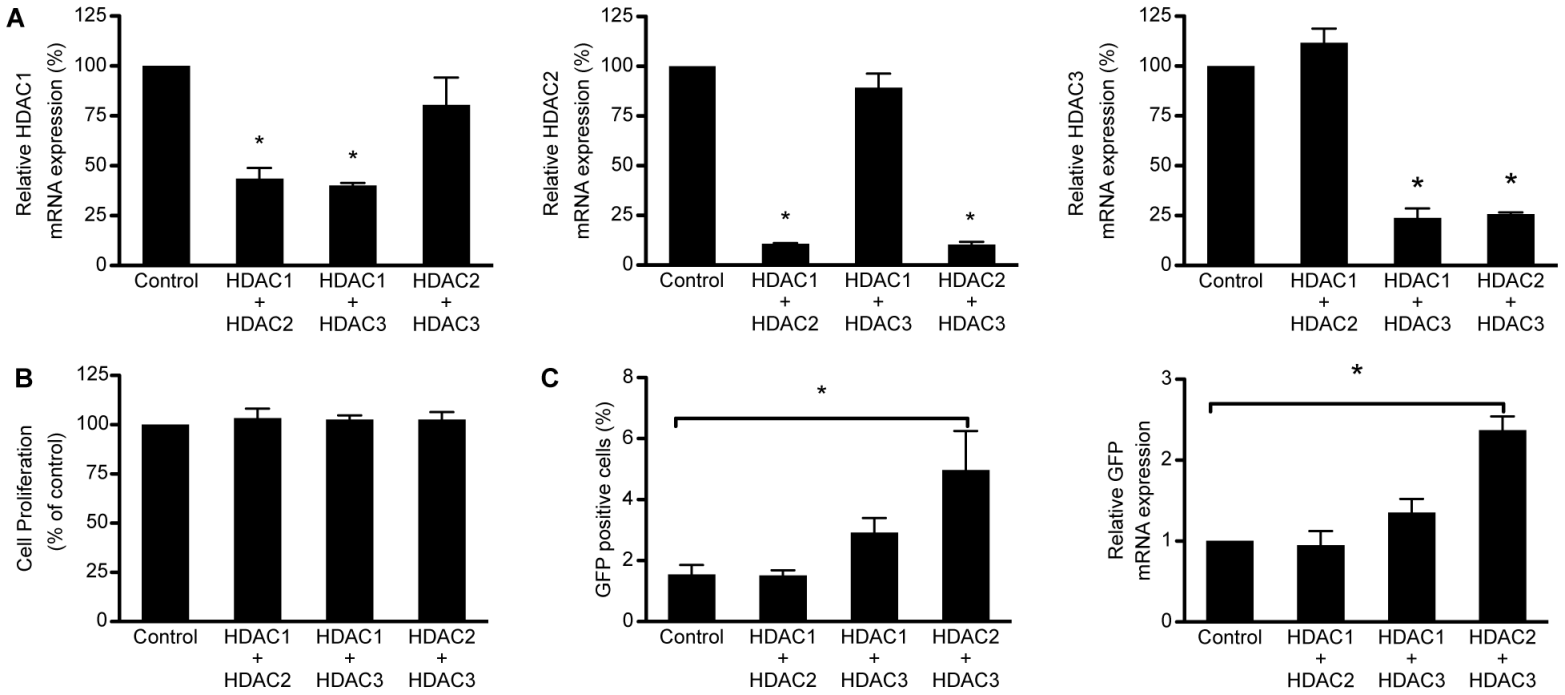
Co-depletion of HDAC2 and HDAC3 significantly increases expression from the HIV-1 promoter. A. Expression of the indicated HDAC mRNAs following shRNA mediated depletion of HDAC1 and −2, HDAC1 and −3, or HDAC2 and −3. The depletion was significant and specific for the targeted HDACs. B. Cell proliferation and viability of 2D10 cells that were transduced with shRNAs targeting HDAC1 and −2, HDAC1 and −3, or HDAC2 and −3. No significant changes in cell proliferation and viability were observed following co- depletion of these HDACs as compared to cells that were transduced with the scrambled shRNA control. C. Expression of GFP from the HIV-1 promoter following depletion of HDAC2 and −3 increased compared to the control cells transduced with a scrambled shRNA. However, depletion of HDAC1 and −2 or HDAC1 and −3 did not have a significant effect on GFP expression. This same result was seen when GFP mRNA expression was directly measured. Expression was not significantly altered from baseline following transduction with non-specific shRNA. (*p<0.05).

We previously reported that inhibitors selective for HDAC1, −2, and −3 are potent inducers of HIV-1 expression [21]. Therefore, to determine if dual inhibition of a pair of these HDACs increased the induction of HIV-1 expression, compared to inhibition of HDAC3 alone, we evaluated the effects of combined shRNA-mediated HDAC knockdown on latent HIV-1 expression in 2D10 cells. The targeted HDAC mRNA expression levels following combination knockdowns were similar to those obtained following individual knockdowns at 96 hours after transduction (compare Figs. 1A and 2A). Cell viability was not affected at 96 hours following combination HDAC depletion when compared to the scrambled shRNA control (Fig. 2B). Depletion of HDAC2 and −3 resulted in a significant increase in the percent of cells expressing HIV-1 LTR driven GFP protein and in HIV-1 driven GFP mRNA expression over the cells that were transduced with the scrambled shRNA (Fig. 2C). However, this increase was not significantly different from the increase observed following depletion of HDAC3 alone. The combined knockdown of HDAC1 and −3 resulted in a modest, but not significant, increase in the percent of cells expressing GFP protein and HIV-1 driven mRNA expression over cells that were transduced with the scrambled control (Fig. 2C). Similar to single knockdown of HDAC1 or −2, we did not observe an induction of GFP protein or mRNA expression when HDAC1 and −2 were inhibited together. Therefore, depletion of HDAC1 or −2 in combination with HDAC3 does not enhance the inducing effects observed following depletion of HDAC3 alone.

### Combined knockdown of HDAC1, −2, and −3 induces modest expression from the HIV-1 LTR

**Figure 3:**
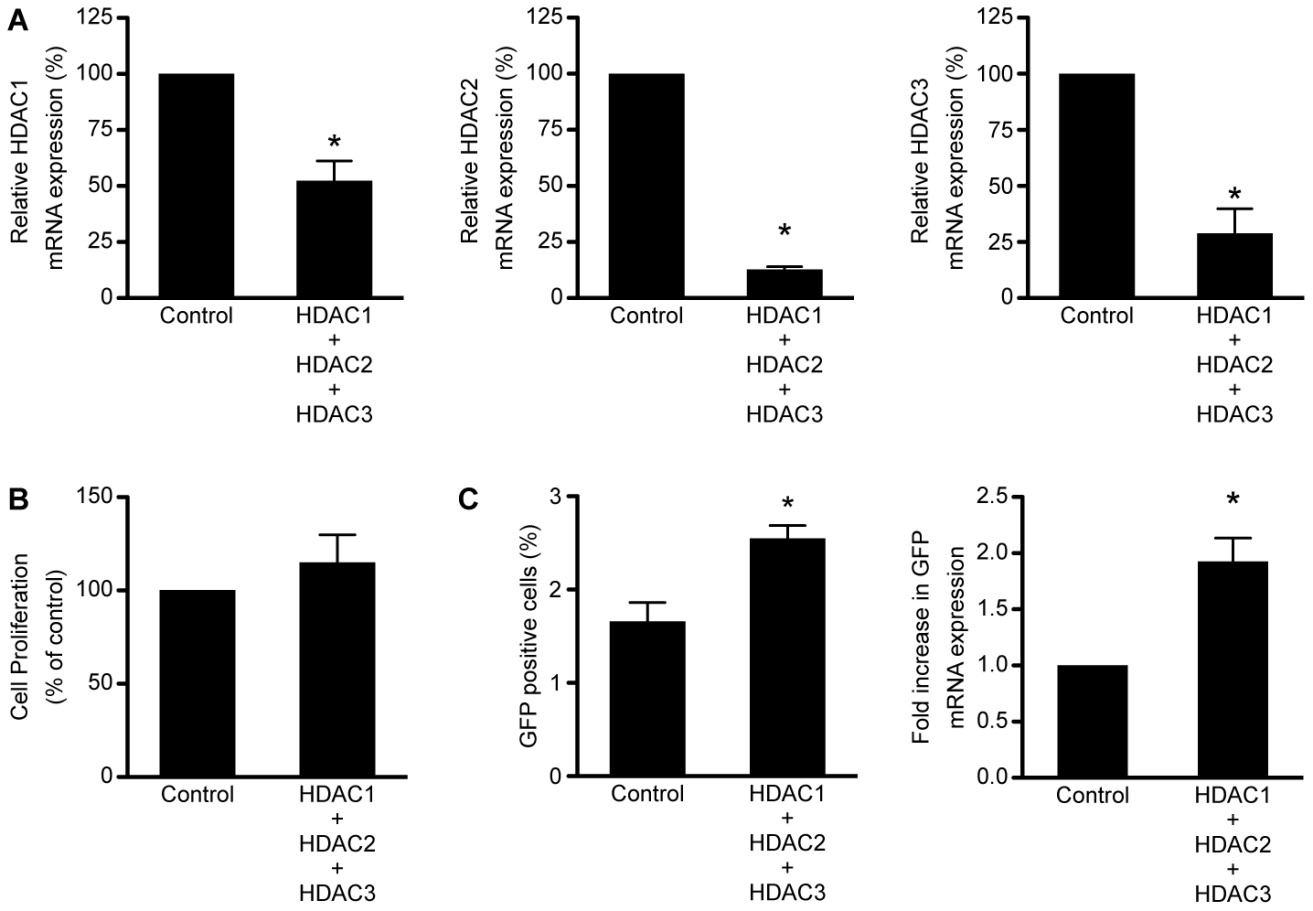
Depletion of HDAC1, −2, and −3 significantly increases expression from the HIV-1 promoter. A. Fold change of HDAC1, −2, and −3 mRNA expression as compared to 2D10 cells transduced with the scrambled shRNA control. A significant reduction in the mRNA expression of all three HDACs was observed. B. Cell viability and proliferation as a percentage of the 2D10 cells transduced with the scrambled shRNA control. Depletion of HDAC1, −2, and −3 did not significantly affect cell viability and proliferation. C. A significant increase in the percentage of cells expressing GFP protein from the HIV-1 promoter was observed following depletion of HDAC1, −2, and −3. Furthermore, a significant increase in expression of GFP mRNA from the HIV-1 promoter was observed following depletion of HDAC1, −2, and −3. (*p<0.05).

As we have previously observed induction of HIV-1 expression in 2D10 cells with inhibitors that selectively target HDAC1, −2, and −3, it is possible that optimal induction of HIV-1 may require simultaneous inhibition of all three HDACs [21]. Thus, we tested the effects of combined knockdown of HDAC1, −2, and −3 on HIV-1 expression using the shRNAs described above. HDAC mRNA levels were reduced at 96 hours after transduction with HDAC shRNAs when compared to transduction with the scrambled shRNA control (Fig. 3A). Concurrent knockdown of HDAC1, −2, and −3 by simultaneously transducing the three individual shRNAs that were used in the single knockdowns did not affect cellular viability at 96 h post-transduction when compared to the scrambled shRNA control (Fig. 3B). A significant increase in the percent of cells expressing GFP protein and in mRNA expression from the HIV-1 promoter was observed in cells depleted of HDAC1, −2, and −3 as compared to the scrambled shRNA control (Fig. 3C). However, as with the dual combination knockdowns, the amount of expression induced by all three HDACs was not significantly more than that observed following depletion of HDAC3 alone. Therefore, the significant increase observed following depletion of all three HDACs over the scrambled shRNA control condition is likely due to the effects of HDAC3 depletion.

### Enzymatic inhibition of HDACs potentiates the effects of HDAC3 depletion

**Figure 4:**
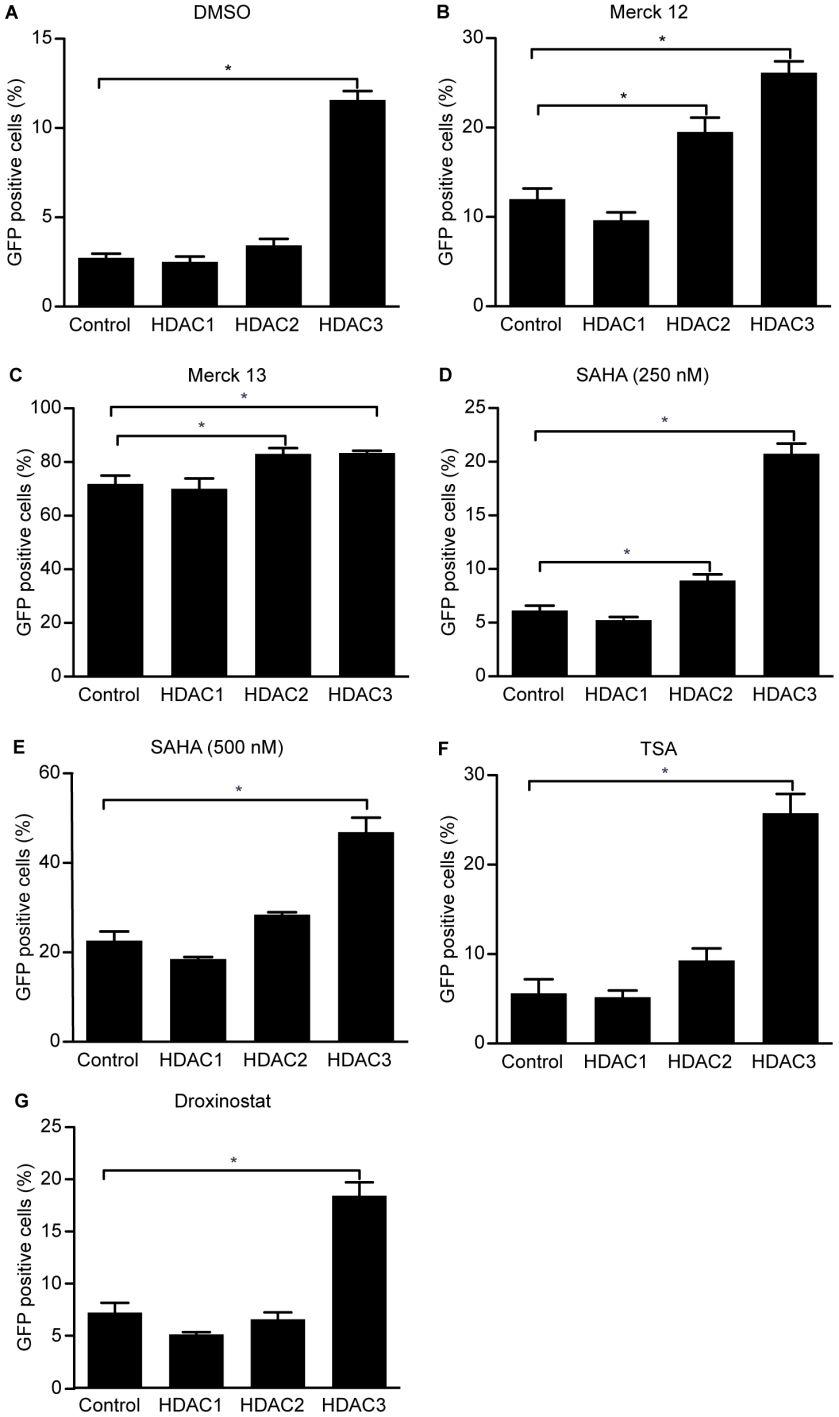
Chemical inhibition of HDACs following depletion of HDAC3 significantly increases expression from the HIV-1 promoter. A. 2D10 cell that had been depleted of HDAC1, −2, or −3 or had been infected with the scrambled control lentivirus were exposed to 0.015% DMSO as the vehicle control for 24 hours. B. Chemical inhibition of HDAC1 and −2 using Mrk 12 (20 µM) does not result in a significant increase in the percentage of 2D10 cells expressing GFP following depletion of HDAC1 or −2. However, similar to depletion of HDAC3 alone, a significant increase in the percent of cells expressing GFP was observed when Mrk 12 was added to cells depleted of HDAC3. C. Chemical inhibition of HDAC1, −2 and −3 with Mrk 13 (200 nM) resulted in a significant increase in the percent of GFP positive 2D10 cells in cells depleted of HDAC2 or −3, but not HDAC1. D. A submaximal (250 nM) concentration of SAHA resulted in a significant increase in the percent of cells expressing GFP following depletion of HDAC2 and −3. E. A maximal concentration of SAHA (500 nM) resulted in a significant increase in the percent of cells expressing GFP in cells depleted of HDAC3. However, in D. or E. depletion of HDAC1 did not increase the percent of cells expressing GFP. F. Chemical inhibition of HDACs with the non-selective inhibitor TSA (25 nM) resulted in a significant increase in the percent of GFP expressing 2D10 cells in cells depleted of HDAC3, but not HDAC1 or HDAC2. G. Chemical inhibition of HDAC3, −6, and −8 with the selective HDAC inhibitor Droxinostat (2 µM) resulted in a significant increase in the percent of 2D10 cells expressing GFP in cells that had been depleted of HDAC3 but not HDAC1 or −2. (*p<0.05).

Because the depletion of the HDACs was not complete when using the shRNAs to deplete the HDAC protein, we next used both selective and global HDAC inhibitors to enzymatically inhibit the remaining HDAC activity following targeted shRNA-mediated depletion. HIV-1 LTR expression in the presence of the vehicle (DMSO) used for these inhibitors is low, and the effect of HDAC3 depletion was significant (Fig. 4A). In the presence of selective inhibition of HDAC1 and −2 with 20 µM of the drug Mrk12 [21], depletion of HDAC1 did not significantly affect the percentage of cells expressing GFP from the HIV-1 promoter (Fig. 4B). When Mrk12 was added to cells that were depleted of HDAC2 there was a modest increase in the percentage of cells expressing GFP from the HIV-1 promoter over cells treated with the scrambled shRNA control and Mrk12. However, when Mrk12 was added to cells that were depleted of HDAC3 a 3-fold increase in the percentage of cells expressing GFP from the HIV-1 promoter was observed over cells treated with the scrambled shRNA control and Mrk12 (Fig. 4B). Furthermore, 200 nM of Mrk13 an inhibitor selective for HDAC1, −2, and −3 [21] activated GFP expression by itself, but a further increase in the percent of cells expressing GFP from the HIV-1 promoter was seen in cells that were depleted of HDAC2 and −3 but not HDAC1 or transduced with the scrambled shRNA control (Fig. 4C).

Next, we used the HDAC inhibitor suberoylanilide hydroxamic acid (SAHA or vorinostat), which is selective for HDAC1, −2, −3, and −6 to partially (at 250 nM) or fully (at 500 nM) inhibit HDAC1, −2, and −3. Similar to the results found with Mrk12 and 13, the suboptimal concentration of SAHA induced a significant increase in the percentage of GFP expressing cells in cells that were depleted of HDAC2 and −3 but not HDAC1 (Fig. 4D). When the maximal concentration of SAHA was combined with depletion of HDAC1, −2, or −3, we observed a significant increase in the percent of cells expressing GFP from the HIV-1 promoter in the cells depleted of HDAC3, but not HDAC1 or −2, over 2D10 cells treated with the scrambled shRNA control and SAHA (Fig. 4E). Furthermore, inhibition with a suboptimal concentration (25 nM) of the non-selective HDAC inhibitor TSA also significantly enhanced the effect of HDAC3 depletion (Fig. 4F). As the percentage of cells expressing GFP from the HIV-1 promoter increased somewhat in three of the conditions in cells depleted of HDAC2 (Fig. 4B–D), we cannot rule out a contributory role for HDAC2 in repression of HIV-1 transcription. However, expression from the HIV-1 promoter increased in combination with all of the tested drugs in cells depleted of HDAC3, indicating a consistent role for HDAC3 in mediating HIV-1 LTR repression.

To determine whether further inhibition of HDAC3 without inhibition of HDAC1 or −2 was sufficient to induce transcription from the HIV-1 LTR we used Droxinostat, an HDAC inhibitor that selectively inhibits HDAC3, −6, and −8 [36]. Droxinostat (2 µM) was used to inhibit HDAC3 activity after depletion of HDAC1, −2, or −3. Again, depletion of HDAC1 or −2 with inhibition of HDAC3 did not increase the percent of cells with LTR driven GFP protein expression observed over Droxinostat treatment of the cells transduced with the scrambled shRNA (Fig. 4G). However, inhibiting the enzymatic activity of the remaining HDAC3 enzyme with Droxinostat following HDAC3 depletion induced a significant increase in the percent of cells expressing GFP protein from the HIV-1 LTR compared to cells that were transduced with the scrambled control and treated with Droxinostat (Fig. 4G). Altogether, these results support the above results, indicating that inhibition of HDAC3 is key for reactivation of latent HIV-1, and suggest a weaker, contributory role for HDAC2.

### JQ1 enhances HIV-1 transcription in HDAC3 depleted cells

**Figure 5:**
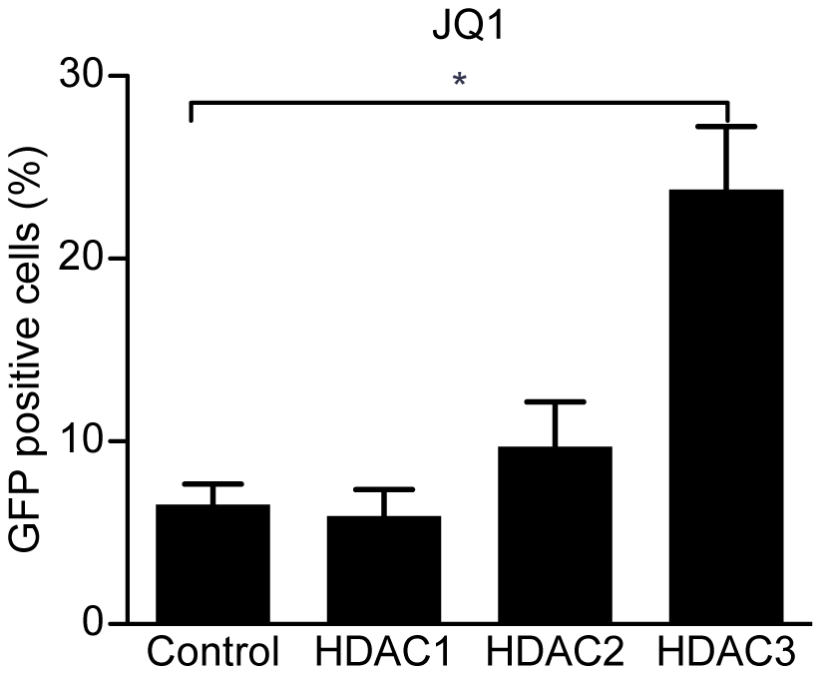
JQ1 enhances the effects of HDAC3 depletion. A significant increase in GFP protein expression from the HIV-1 promoter was observed in 2D10 cells that had been depleted of HDAC3 and had been treated with JQ1 (50 nM) for 24 hours over cells that were transduced with the scrambled shRNA control and treated with JQ1. However, no effect was observed in cells depleted of HDAC1 or HDAC2. (*p<0.05).

It is not clear that HDAC inhibitors can induce a sufficient level HIV-1 expression *in vivo* to allow clearance of latent infection, and so combinatorial approaches to disrupt latency have been proposed [18], [19]. JQ1 is an inhibitor of bromodomain proteins that is capable of inducing expression of quiescent HIV-1 proviral promoters [35], [37]. We sought to determine whether JQ1 could also enhance the effect of HDAC3 depletion. To this end, cells that had been depleted of HDAC1, −2, or −3 were treated with 50 nM JQ1 overnight. Similar to the results from the HDAC inhibitor studies, we found that LTR expression induced by JQ1 was enhanced by depletion of HDAC3, but not HDAC1 or −2 (Fig. 5).

### HDAC3 selective inhibition induces expression of HIV

**Figure 6:**
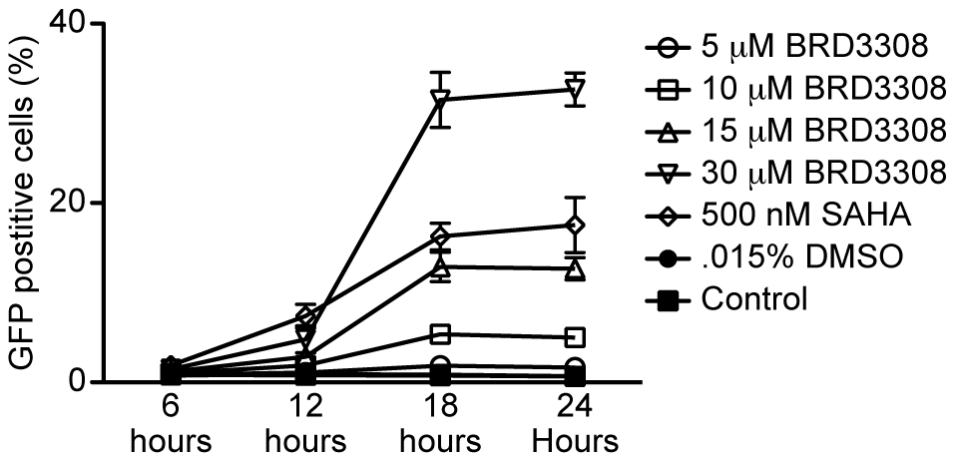
An HDAC3 selective inhibitor induces HIV-1 expression from 2D10 cells. 2D10 cells were treated with BRD3308 at the indicated concentration for 6 hours, 12 hours, 18 hours, or 24 hours and collected for analysis at 24 hours. The percent of 2D10 cells expressing GFP was measured using flow cytometry. HIV-1 expression was observed with 10 µM of BRD3308 and higher after 12 hours of treatment.

BRD3308 is a derivative of the *ortho*-aminoanilide HDAC inhibitor CI-994 and was developed to be highly selective for inhibition of HDAC3 with an IC_50_ value that is 23-fold lower for HDAC3 than for HDAC1 or −2 (Table 1). To determine whether enzymatic inhibition of HDAC3 was sufficient to induce transcription of HIV-1 in a cell line model of HIV, 2D10 cells were exposed to either 5 µM, 10 µM, 15 µM, or 30 µM of BRD3308 for 6, 12, 18, or 24 hours, and LTR-driven GFP expression was monitored by flow cytometry. By 12 hours at concentrations of 10 µM and greater an increase in HIV-1 expression was observed in the 2D10 cell line (Fig. 6).

**Table 1:** IC50 values of BRD3308 and SAHA for HDACs 1–9.

	HDAC Profile	HDAC IC50 (µM) without preincubation								
		HDAC1	HDAC2	HDAC3	HDAC4	HDAC5	HDAC6	HDAC7	HDAC8	HDAC9
**BRD338**	3	1.26	1.34	0.054	>33	>33	>33	>33	>33	>33
**SAHA**	1–3, 6	0.004	0.011	0.003	>33	8.75	0.002	>33	1.02	>33

### Inhibition of HDAC3 induces outgrowth of HIV-1 from patient cells

Given the limitation of clonal cell line models of HIV-1 latency, and the recently recognized limitations of polyclonal primary cell models of HIV-1 latency [34], we wished to directly test the role of HDAC 3 inhibition through the use of the gold-standard quantitative viral outgrowth assay using resting CD4+T cells isolated from aviremic patients on ART [1], [11], [21], [29]. Attempts to accomplish this by transduction of patient's cells with siRNAs targeting HDACs 1–3 were not inconsistent with a role for HDAC3, and a contributory role for HDAC2, in the maintenance of HIV-1 latency. However, the studies were limited by incomplete HDAC knockdown and upregulation of CD69 on resting CD4+ T cells induced by the nucleofection procedure, and limited induction of viral outgrowth by knockdown of HDAC2 or −3 (data not shown).

**Figure 7:**
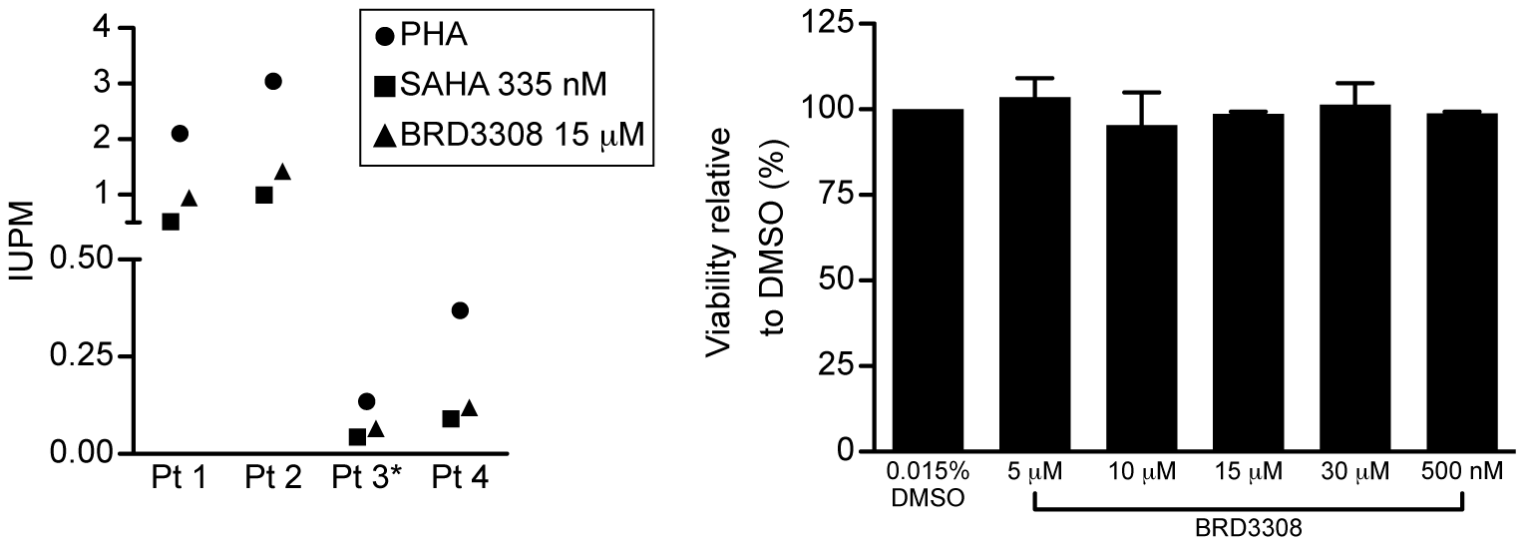
Inhibition of HDAC3 induces outgrowth of HIV-1 from latently infected patient cells ex vivo. A. HIV-1 outgrowth from latently infected CD4+ T cells following overnight exposure to 15 µM BRD3308 or 335 nM SAHA. The frequency of viral outgrowth following mitogen stimulation of HDAC inhibitor exposure is expressed as infected cells per million resting CD4+ T cells (IUPM). In four patients (Pt 1, Pt 2, Pt 3, and Pt 4) exposure to BRD3308 induced a comparable frequency of outgrowth to that observed following exposure to SAHA. *IUPM for PHA for Pt.3 is obtained from three prior assays; all other assays are contemporaneous. B. PBMCs were exposed to 5, 10, 15, or 30 µM of BRD3308, and viability was measured. The results are expressed as the viability relative to the control DMSO condition. BRD3308 does not significantly affect viability of PBMCs.

We therefore compared the induction of viral outgrowth following the exposure of patients' resting CD4+ T cells to the HDAC3 selective inhibitor BRD3308 or the Class I HDAC inhibitor SAHA. Cells from four patients (Pt 1, Pt 2, Pt 3, and Pt 4) were exposed to 15 µM of BRD3308 or 335 nM of SAHA overnight. In all four patients, 15 µM of BRD3308 induced a similar or greater amount of viral outgrowth as exposure to SAHA (Fig. 7A). Furthermore, PBMCs exposed to 5 µM, 10 µM, 15 µM, or 30 µM BRD3308 for 24 hours did not show a significant decrease in viability when compared to PBMCs exposed to the vehicle control (0.015% DMSO) (Fig. 7B). Additionally, the percent of cells expressing the T cell activation markers CD69, CD25, and HLA-DR did not increase following treatment with 15 µM of BRD3308 (data not shown). This data demonstrates that exposure to an HDAC3 selective inhibitor allows the recovery of latent HIV-1 from patient cells at a similar frequency as the selective Class I HDAC inhibitor SAHA without decreasing cell viability or inducing cell activation, in contrast to the absence of viral induction observed following exposure to an inhibitor specific for HDAC1 and −2 [38].

## Discussion

Selective inhibitors that target HDAC1, −2, and −3, but not those that only inhibit HDAC1 and −2, are potent inducers of HIV-1 transcription [18], [21]. In an effort to determine the minimal HDAC-isoform(s) inhibition that is necessary for optimal induction of latent HIV-1, we employed an shRNA-mediated strategy to deplete individual and combinations of the class I HDACs, HDAC1, −2, and −3, in the 2D10 T cell line model of quiescent HIV-1. We found that isolated depletion of HDAC3 expression resulted in a statistically significant induction of LTR driven GFP protein expression in 2D10 cells (Fig. 1D). Furthermore, inhibition of HDAC3 in combination with HDAC1, −2, or both HDAC1 and −2 induced a significant increase in expression from the HIV-1 promoter, but not significantly more than depletion of HDAC3 alone. These findings support our previous studies on the effects of selective HDAC inhibitors and further define the minimal HDAC inhibition that is required for reactivation of latent HIV-1 [21].

It worth noting that the rate of knockdown achieved by the individual HDAC shRNAs in this study ranged from 48% to 95% (Figs. 1A, 2A, 3A, and 5A); thus, there was not a complete elimination of any of the HDACs targeted. Because HDAC1, −2, and −3 are all known to associate with the HIV-1 LTR [21], it is possible that some combination of these HDACs may remain to regulate the viral promoter following knockdown with shRNA. Furthermore, transduction with shRNAs results in a slow depletion of protein expression. In contrast, chemical HDAC enzymatic inhibition occurs rapidly and persists until the drug is cleared.

To address these concerns, we depleted 2D10 cells of HDAC1, −2, or −3 and used small molecules inhibitors to block the remaining HDAC activity. We found that in cells depleted of HDAC3, chemical inhibition of the remaining HDAC activity enhanced expression of GFP from the HIV-1 LTR for all of the drugs studied. Furthermore, a modest increase in expression from the HIV-1 LTR was also observed in cells that were depleted of HDAC2 when cells were exposed to the HDAC inhibitors MRK12 and MRK13, and to SAHA, indicating that HDAC2 may also have a contributory role in the maintenance of HIV-1 latency. Importantly, no change in expression from the HIV-1 promoter was observed in cells depleted of HDAC1. Altogether, the consistent increase in expression from the HIV-1 promoter that was observed in cells depleted of HDAC3 indicates that HDAC3 is a key factor in repression of HIV-1 expression.

Keedy et al. used siRNAs to deplete the HeLa P4/R5 cell line model of latent HIV-1 of HDAC1, −2, or −3 and observed that depletion of HDAC2 alone induced LacZ expression from the HIV-1 promoter and that depletion of HDAC3, but not HDAC2, in conjunction with 1 uM of TSA significantly induced expression over treatment with TSA alone. It is interesting that no increase in HIV-1 driven LacZ expression was observed following HDAC3 depletion alone in this study, which is in contrast to the results from this study. This discrepancy may be attributable to differences in the level of knockdown, differences between the models or with the different methodologies that were used. HeLa cells are an epithelial cell line that is physiologically different from latently infected CD4+ T cells. Jurkat cells, such as the 2D10 cells used in this study, are CD4+ lymphoid cells more closely related to CD4+ T cells most commonly infected by HIV-1 *in vivo*. However, although these two cell lines and the studies conducted in them are very different, it is interesting that in both studies HDAC2 and −3, but not HDAC1, were found to be important in expression from the HIV-1 promoter. In another study, Huber et al. depleted, J89GFP cells (Jurkat cell line that contains GFP under the control of the HIV-1 promoter) of HDAC1, −2, or both and found no difference in the percent of GFP positive cells compared to the control siRNA condition, which is similar to the results found in this study when HDAC1 or −2 were depleted individually or in combination. The work in this study further extends the finding from these previous studies to an additional cell line and provides additional evidence that targeting HDAC3 and possibly HDAC2, but not HDAC1, can be used to induce expression from the HIV-1 promoter.

Huber et al. demonstrated that Droxinostat, an inhibitor reported to target HDAC3, −6, and −8, activated transcription from a cell model of HIV-1 latency [36]. However, as other data finds that the IC50s of Droxinostat are 16.9 µM (HDAC3), 2.47 µM (HDAC 6), and 1.46 µM (HDAC 8) [39], the primacy of HDAC3 inhibition was unclear. In this study, treating 2D10 cells depleted of HDAC3 with 2 µM of Droxinostat resulted in a significant increase in GFP expression from the HIV-1 LTR over cells transduced with the scrambled shRNA and treated with Droxinostat (Fig. 4E). A significant increase in LTR-driven GFP expression was detected with 2 µM of Droxinostat, which is the IC_50_ of this drug for HDAC3 and is 2.5 fold below the IC_50_ value for HDAC8 and 30 fold below the IC_50_ for HDAC1 [36]. These results further support the above results indicating that targeting HDAC3 alone may be sufficient to induce transcription from quiescent HIV-1 proviruses.

JQ1 is an inhibitor of Brd4 that has been demonstrated to be able to induce HIV-1 transcription [35], [37]. In this study, it was demonstrated that treating HDAC3 depleted 2D10 cells with JQ1 significantly increased expression from the HIV-1 promoter over the amount of expression observed following HDAC3 depletion or treatment with JQ1 only. Therefore, selective inhibition of HDAC3 may have potential for use in combination therapies with Brd4 inhibitors like JQ1.

To reinforce the role of HDAC3, we next tested the effect of BRD3308, which is an HDAC3 selective inhibitor. In addition to inducing expression in the 2D10 cell line, BRD3308 is able to induce outgrowth of HIV-1 from latently infected patient cells *ex vivo* comparably to the HDAC inhibitor SAHA (Fig. 7A). These results indicate that more selective inhibition of HDAC3 may be sufficient to reverse latency.

HDAC1, −2, and −3 have been demonstrated to physically associate with the HIV-1 LTR [40], and inhibition of these HDACs with small molecule HDAC inhibitors is associated with increased acetylation of histones at the HIV-1 LTR [41]. Therefore, it is interesting that depletion of HDAC3, but not HDAC1 or −2, activates transcription from the HIV-1 promoter. HDAC3 is unique from HDAC1 and −2 in several aspects, which may explain its unique role in the maintenance of HIV-1 latency. HDAC1 and −2 are 85% similar to each other; however, HDAC3 is only 53% and 52% similar to HDAC1 and −2, respectively. Furthermore, HDAC3 contains a unique amino acid sequence at its carboxy terminus that is not found in HDAC1 or −2 [42]. In addition to its nuclear localization signal, HDAC3 contains cytoplasmic and cell membrane-targeting regions that are not found in HDAC1 and −2 [42]. HDAC2 and −3 are located in both the cytoplasm and nucleus in resting and activated CD4+ T cells from HIV-1 infected aviremic patients [40]. In contrast, HDAC1 is only found in the nucleus [40]. In addition to affecting localization, these sequence differences may also account for HDAC3's unique complex association. HDAC3 is associated with the NCoR and SMRT complexes, while HDAC1 and −2 are associated with the CoREST, NuRD, REST, and Sin complexes [43], [44]. NCoR has been demonstrated to bind to the HIV-1 LTR in monocyte-derived macrophages [45]. Furthermore, we find NCoR at the HIV-1 LTR in the J89 Jurkat cell line model of HIV-1 latency (Barton and Margolis, data not shown). The association of NCoR with the HIV-1 promoter indicates that HDAC3 may be mediating its effects through the NCoR complex. Therefore, the differences in effects observed following individual depletion of HDAC1, −2, and −3 may be due to the differences in complex association, sequence diversity or expression.

Although HDAC3 depletion and inhibition resulted in consistent activation of quiescent HIV, a putative contribution of HDAC2 was observed when it was depleted in conjunction with HDAC3. Of the HDACs that are found at the HIV-1 promoter, HDAC1 and −3 have the most highly expressed mRNAs and are expressed five and eight times more than that of HDAC2, respectively [40]. Therefore, the minimal role of HDAC2 in reactivation of latent HIV-1 may be associated with its low level of expression relative to the other two HDACs.

We sought to determine the minimal set of HDAC-isoform(s) whose inhibition is necessary to induce latent HIV-1 expression. Selective HDAC inhibitors targeting HDAC1 and −2 were not sufficient to induce latent HIV-1 expression [21]. Furthermore, selective chemical inhibition of HDAC3 was sufficient to induce transcription in both the cell line model of HIV-1 latency and *ex vivo* from latently infected patient cells. In this study, selective targeting of HDAC3 was demonstrated to be sufficient to induce expression from the HIV-1 promoter.

Selective HDAC inhibitors that target a limited number of the class I HDACs have potential as anti-latency therapies in HIV-1 infection with fewer host toxicities as selective inhibitors may have reduced off-target effects. We previously demonstrated that inhibitors that selectively target HDAC1, −2, and −3—but not HDAC1 and −2 alone—are potent inducers of latent HIV-1 [21]. In this study, we evaluated whether a more selective induction of HIV-1 could be achieved using a shRNA-mediated strategy of HDAC knockdown in the 2D10 cell line model of HIV latency. Our results indicate that depletion or inhibition of HDAC3 significantly induces expression from the HIV-1 promoter. However, a potential contributory role of HDAC2 cannot be ruled out, and it appears that molecules that inhibit HDAC3 (and perhaps HDAC2) but avoid HDAC1 inhibition might mediate maximal HIV-1 LTR induction. Overall, we conclude that further studies are needed to optimally design HDAC inhibitors for use in anti-latency strategies and to determine if it is clinically preferable to specifically target HDAC3 in the hope of avoiding clinical toxicities or to target both HDAC2 and −3 in the hope of optimally inducing a broad array of latent HIV-1 genomes.

## References

[1] Finzi D, Hermankova M, Pierson T, Carruth LM, Buck C, et al (1997) Identification of a reservoir for HIV-1 in patients on highly active antiretroviral therapy. Science 278: 1295–1300. 9360927 10.1126/science.278.5341.1295

[2] Finzi D, Blankson J, Siliciano JD, Margolick JB, Chadwick K, et al (1999) Latent infection of CD4+ T cells provides a mechanism for lifelong persistence of HIV-1, even in patients on effective combination therapy. Nat Med 5: 512–517. 10229227 10.1038/8394

[3] Chun TW, Engel D, Berrey MM, Shea T, Corey L, et al (1998) Early establishment of a pool of latently infected, resting CD4(+) T cells during primary HIV-1 infection. Proc Natl Acad Sci U S A 95: 8869–8873. 9671771 10.1073/pnas.95.15.8869 PMC21169

[4] UNAIDS (2008) 2008 Report on the global AIDS epidemic.

[5] Ganchi PA, Sun SC, Greene WC, Ballard DW (1992) I kappa B/MAD-3 masks the nuclear localization signal of NF-kappa B p65 and requires the transactivation domain to inhibit NF-kappa B p65 DNA binding. Mol Biol Cell 3: 1339–1352. 1493333 10.1091/mbc.3.12.1339 PMC275704

[6] Garriga J, Peng J, Parreno M, Price DH, Henderson EE, et al (1998) Upregulation of cyclin T1/CDK9 complexes during T cell activation. Oncogene 17: 3093–3102. 9872325 10.1038/sj.onc.1202548

[7] Okamura H, Aramburu J, Garcia-Rodriguez C, Viola JP, Raghavan A, et al (2000) Concerted dephosphorylation of the transcription factor NFAT1 induces a conformational switch that regulates transcriptional activity. Mol Cell 6: 539–550. 11030334 10.1016/s1097-2765(00)00053-8

[8] Williams SA, Kwon H, Chen LF, Greene WC (2007) Sustained induction of NF-kappa B is required for efficient expression of latent human immunodeficiency virus type 1. J Virol 81: 6043–6056. 17376917 10.1128/JVI.02074-06 PMC1900291

[9] Swiggard WJ, Baytop C, Yu JJ, Dai J, Li C, et al (2005) Human immunodeficiency virus type 1 can establish latent infection in resting CD4+ T cells in the absence of activating stimuli. J Virol 79: 14179–14188. 16254353 10.1128/JVI.79.22.14179-14188.2005 PMC1280214

[10] Lassen K, Han Y, Zhou Y, Siliciano J, Siliciano RF (2004) The multifactorial nature of HIV-1 latency. Trends Mol Med 10: 525–531. 15519278 10.1016/j.molmed.2004.09.006

[11] Ylisastigui L, Archin NM, Lehrman G, Bosch RJ, Margolis DM (2004) Coaxing HIV-1 from resting CD4 T cells: histone deacetylase inhibition allows latent viral expression. AIDS 18: 1101–1108. 15166525 10.1097/00002030-200405210-00003

[12] Choudhary SK, Archin NM, Margolis DM (2008) Hexamethylbisacetamide and disruption of human immunodeficiency virus type 1 latency in CD4(+) T cells. J Infect Dis 197: 1162–1170. 18419522 10.1086/529525

[13] Coull JJ, Romerio F, Sun JM, Volker JL, Galvin KM, et al (2000) The human factors YY1 and LSF repress the human immunodeficiency virus type 1 long terminal repeat via recruitment of histone deacetylase 1. J Virol 74: 6790–6799. 10888618 10.1128/jvi.74.15.6790-6799.2000 PMC112196

[14] Marban C, Suzanne S, Dequiedt F, de Walque S, Redel L, et al (2007) Recruitment of chromatin-modifying enzymes by CTIP2 promotes HIV-1 transcriptional silencing. The EMBO journal 26: 412–423. 17245431 10.1038/sj.emboj.7601516 PMC1783449

[15] Gregoretti IV, Lee YM, Goodson HV (2004) Molecular evolution of the histone deacetylase family: functional implications of phylogenetic analysis. J Mol Biol 338: 17–31. 15050820 10.1016/j.jmb.2004.02.006

[16] Archin NM, Espeseth A, Parker D, Cheema M, Hazuda D, et al (2009) Expression of latent HIV induced by the potent HDAC inhibitor suberoylanilide hydroxamic acid. AIDS Res Hum Retroviruses 25: 207–212. 19239360 10.1089/aid.2008.0191 PMC2853863

[17] Edelstein LC, Micheva-Viteva S, Phelan BD, Dougherty JP (2009) Short communication: activation of latent HIV type 1 gene expression by suberoylanilide hydroxamic acid (SAHA), an HDAC inhibitor approved for use to treat cutaneous T cell lymphoma. AIDS Res Hum Retroviruses 25: 883–887. 19689202 10.1089/aid.2008.0294 PMC2828260

[18] Savarino A, Mai A, Norelli S, El Daker S, Valente S, et al (2009) “Shock and kill” effects of class I-selective histone deacetylase inhibitors in combination with the glutathione synthesis inhibitor buthionine sulfoximine in cell line models for HIV-1 quiescence. Retrovirology 6: 52. 19486542 10.1186/1742-4690-6-52 PMC2697151

[19] Reuse S, Calao M, Kabeya K, Guiguen A, Gatot JS, et al (2009) Synergistic activation of HIV-1 expression by deacetylase inhibitors and prostratin: implications for treatment of latent infection. PloS one 4: e6093. 19564922 10.1371/journal.pone.0006093 PMC2699633

[20] Archin N, Liberty AL, Kashuba AD, Choudhary SK, Kuruc J, et al (2012) Administration of vorinostat disrupts HIV-1 latency in patients on antiretroviral therapy. Nature 10.1038/nature11286 PMC3704185 22837004

[21] Archin NM, Keedy KS, Espeseth A, Dang H, Hazuda DJ, et al (2009) Expression of latent human immunodeficiency type 1 is induced by novel and selective histone deacetylase inhibitors. AIDS 23: 1799–1806. 19590405 10.1097/QAD.0b013e32832ec1dc PMC3809117

[22] Jiang G, Espeseth A, Hazuda DJ, Margolis DM (2007) c-Myc and Sp1 contribute to proviral latency by recruiting histone deacetylase 1 to the human immunodeficiency virus type 1 promoter. J Virol 81: 10914–10923. 17670825 10.1128/JVI.01208-07 PMC2045540

[23] Tyagi M, Karn J (2007) CBF-1 promotes transcriptional silencing during the establishment of HIV-1 latency. Embo J 26: 4985–4995. 18007589 10.1038/sj.emboj.7601928 PMC2140115

[24] Williams SA, Chen LF, Kwon H, Ruiz-Jarabo CM, Verdin E, et al (2006) NF-kappaB p50 promotes HIV latency through HDAC recruitment and repression of transcriptional initiation. Embo J 25: 139–149. 16319923 10.1038/sj.emboj.7600900 PMC1356344

[25] Kutsch O, Benveniste EN, Shaw GM, Levy DN (2002) Direct and quantitative single-cell analysis of human immunodeficiency virus type 1 reactivation from latency. Journal of virology 76: 8776–8786. 12163598 10.1128/JVI.76.17.8776-8786.2002 PMC136999

[26] Jordan A, Bisgrove D, Verdin E (2003) HIV reproducibly establishes a latent infection after acute infection of T cells in vitro. The EMBO journal 22: 1868–1877. 12682019 10.1093/emboj/cdg188 PMC154479

[27] Pearson R, Kim YK, Hokello J, Lassen K, Friedman J, et al (2008) Epigenetic silencing of human immunodeficiency virus (HIV) transcription by formation of restrictive chromatin structures at the viral long terminal repeat drives the progressive entry of HIV into latency. J Virol 82: 12291–12303. 18829756 10.1128/JVI.01383-08 PMC2593349

[28] Ferguson BS, Harrison BC, Jeong MY, Reid BG, Wempe MF, et al (2013) Signal-dependent repression of DUSP5 by class I HDACs controls nuclear ERK activity and cardiomyocyte hypertrophy. Proc Natl Acad Sci U S A 110: 9806–9811. 23720316 10.1073/pnas.1301509110 PMC3683796

[29] Archin NM, Espeseth A, Parker D, Cheema M, Hazuda D, et al (2009) Expression of latent HIV induced by the potent HDAC inhibitor suberoylanilide hydroxamic acid. AIDS research and human retroviruses 25: 207–212. 19239360 10.1089/aid.2008.0191 PMC2853863

[30] Katragadda M, Magotti P, Sfyroera G, Lambris JD (2006) Hydrophobic effect and hydrogen bonds account for the improved activity of a complement inhibitor, compstatin. Journal of medicinal chemistry 49: 4616–4622. 16854067 10.1021/jm0603419

[31] Poleshko A, Palagin I, Zhang R, Boimel P, Castagna C, et al (2008) Identification of cellular proteins that maintain retroviral epigenetic silencing: evidence for an antiviral response. J Virol 82: 2313–2323. 18094192 10.1128/JVI.01882-07 PMC2258957

[32] Verhoeven D, Sankaran S, Silvey M, Dandekar S (2008) Antiviral therapy during primary simian immunodeficiency virus infection fails to prevent acute loss of CD4+ T cells in gut mucosa but enhances their rapid restoration through central memory T cells. J Virol 82: 4016–4027. 18272585 10.1128/JVI.02164-07 PMC2292978

[33] Duverger A, Jones J, May J, Bibollet-Ruche F, Wagner FA, et al (2009) Determinants of the establishment of human immunodeficiency virus type 1 latency. J Virol 83: 3078–3093. 19144703 10.1128/JVI.02058-08 PMC2655589

[34] Spina CA, Anderson J, Archin NM, Bosque A, Chan J, et al (2013) An in-depth comparison of latent HIV-1 reactivation in multiple cell model systems and resting CD4+ T cells from aviremic patients. PLoS Pathog 9: e1003834. 24385908 10.1371/journal.ppat.1003834 PMC3873446

[35] Boehm D, Calvanese V, Dar RD, Xing S, Schroeder S, et al (2013) BET bromodomain-targeting compounds reactivate HIV from latency via a Tat-independent mechanism. Cell cycle 12: 452–462. 23255218 10.4161/cc.23309 PMC3587446

[36] Huber K, Doyon G, Plaks J, Fyne E, Mellors JW, et al (2011) Inhibitors of histone deacetylases: correlation between isoform specificity and reactivation of HIV type 1 (HIV-1) from latently infected cells. The Journal of biological chemistry 286: 22211–22218. 21531716 10.1074/jbc.M110.180224 PMC3121366

[37] Banerjee C, Archin N, Michaels D, Belkina AC, Denis GV, et al (2012) BET bromodomain inhibition as a novel strategy for reactivation of HIV-1. Journal of leukocyte biology 92: 1147–1154. 22802445 10.1189/jlb.0312165 PMC3501896

[38] Choudhary SK, Archin NM, Margolis DM (2008) Hexamethylbisacetamide and disruption of human immunodeficiency virus type 1 latency in CD4(+) T cells. The Journal of infectious diseases 197: 1162–1170. 18419522 10.1086/529525

[39] Wood TE, Dalili S, Simpson CD, Sukhai MA, Hurren R, et al (2010) Selective inhibition of histone deacetylases sensitizes malignant cells to death receptor ligands. Mol Cancer Ther 9: 246–256. 20053768 10.1158/1535-7163.MCT-09-0495

[40] Keedy KS, Archin NM, Gates AT, Espeseth A, Hazuda DJ, et al (2009) A limited group of class I histone deacetylases acts to repress human immunodeficiency virus type 1 expression. Journal of virology 83: 4749–4756. 19279091 10.1128/JVI.02585-08 PMC2682072

[41] Lusic M, Marcello A, Cereseto A, Giacca M (2003) Regulation of HIV-1 gene expression by histone acetylation and factor recruitment at the LTR promoter. The EMBO journal 22: 6550–6561. 14657027 10.1093/emboj/cdg631 PMC291826

[42] Yang WM, Yao YL, Sun JM, Davie JR, Seto E (1997) Isolation and characterization of cDNAs corresponding to an additional member of the human histone deacetylase gene family. The Journal of biological chemistry 272: 28001–28007. 9346952 10.1074/jbc.272.44.28001

[43] Guenther MG, Barak O, Lazar MA (2001) The SMRT and N-CoR corepressors are activating cofactors for histone deacetylase 3. Molecular and cellular biology 21: 6091–6101. 11509652 10.1128/MCB.21.18.6091-6101.2001 PMC87326

[44] Hayakawa T, Nakayama J (2011) Physiological roles of class I HDAC complex and histone demethylase. Journal of biomedicine & biotechnology 2011: 129383. 21049000 10.1155/2011/129383 PMC2964911

[45] Hanley TM, Viglianti GA (2011) Nuclear receptor signaling inhibits HIV-1 replication in macrophages through multiple trans-repression mechanisms. Journal of virology 85: 10834–10850. 21849441 10.1128/JVI.00789-11 PMC3187477

